# *Trigonella foenum* (Fenugreek) Induced Apoptosis in Hepatocellular Carcinoma Cell Line, HepG2, Mediated by Upregulation of p53 and Proliferating Cell Nuclear Antigen

**DOI:** 10.1155/2015/914645

**Published:** 2015-10-18

**Authors:** Mahmoud I. M. Khalil, Mohamed M. Ibrahim, Gehan A. El-Gaaly, Ahmed S. Sultan

**Affiliations:** ^1^Zoology Department, Faculty of Science, Alexandria University, Alexandria 21511, Egypt; ^2^Botany and Microbiology Department, Science College, King Saud University, P.O. Box 2455, Riyadh 11451, Saudi Arabia; ^3^Botany and Microbiology Department, Faculty of Science, Alexandria University, Alexandria 21511, Egypt; ^4^Department of Biochemistry, Faculty of Science, Alexandria University, Alexandria 21511, Egypt

## Abstract

Hepatocellular carcinoma (HCC) is one of the most common cancers worldwide and most current therapies are of limited efficacy. *Trigonella foenum* (*Fenugreek*) is a traditional herbal plant with antitumor activity, although the mechanisms of its activity remain unclear. Herein, a crude methanol extract was prepared from Fenugreek seeds (FCE) and its anticancer mechanism was evaluated, using HepG2 cell line. Growth-inhibitory effect and apoptosis induction of HepG2 cells were evidenced by MTT assay, cell morphology alteration, apoptosis enzyme-linked immunosorbent assay, flow cytometric analysis, caspase-3 activity, and expression of p53, proapoptotic protein, Bax, and proliferating cell nuclear antigen (PCNA) after (100∼500 *μ*g/mL) FCE treatment for 48 h. Furthermore, FCE was analyzed by Chromatography-Mass Spectrometry (GC/MS). Our results revealed that FCE treatment for 48 h showed a cytotoxic effect and apoptosis induction in a dose-dependent manner that was mediated by upregulation of p53, Bax, PCNA, and caspase-3 activation in HepG2 cells. GC-MS analysis of FCE showed the presence of fourteen bioactive compounds such as Terpenoids and Flavonoids, including two main constituents with anticancer activity, Squalene and Naringenin (27.71% and 24.05%), respectively. Our data introduced FCE as a promising nontoxic herbal with therapeutic potential to induce apoptosis in HepG2 cells through p53, Bax, and PCNA upregulation in caspase-3 dependent manner.

## 1. Introduction

Hepatocellular carcinoma (HCC) is the sixth most common cancer worldwide, accounting for 9.1% of all cancers and an estimated incidence of 746,000 new cases every year. It is considered to be the third cause of cancer related deaths (692,000 cases). The highest incidence rates of HCC (around 85% of cases) are present in East Asia and sub-Saharan Africa [1]. The prognosis for liver cancer is very poor (overall ratio of mortality to incidence of 0.95), and as such the geographical patterns in incidence and mortality are similar.

In Egypt, there is a growing incidence of HCC (10–120/100,000) [2], which represents the leading cause of death from all other cancer sites [3]. HCC has nearly doubled over the last decade among patients with chronic liver disease (CLD) [4]. HBV and HCV infections are the most important risk factors associated with progression from chronic hepatitis to cirrhosis and, eventually, to HCC. In spite of the advances made in HCC diagnosis treatment, intervention strategies are still badly warranted, including chemopreventive agents that act on the early molecular events of cancer initiation by preventing, delaying, or reversing epithelial-mesenchymal transition, as well as those that act as therapeutic agents for cancer progression [5, 6]. Using chemopreventive agents that have low toxicity and high efficiency in inhibiting tumor growth and are involved in decrease of carcinogenic agents effects is very promising target for cancer therapy [7].

Recently, increasing attention has been focused to identify the naturally occurring anticancer agents, particularly those present in dietary and medicinal plants due to their bioactive substances [8, 9]. Most of these bioactive substances exert their cancer chemotherapeutic activity by modulating signal transduction pathways that are involved in cell cycle progression, proliferation, and triggering apoptotic cell death. In addition, induction of tumor suppressor genes in tumor cells has become promising therapeutic indicator for tumor treatment response in employing a plant derived-bioactive substance to decrease breast cancer mortality.

In many developed countries herbal medicines are achieving attractiveness as alternative and courtesy therapies [10]. Some of the plants are used as food or medicine. These plants display varied kinds of biological and pharmacological activities. Fenugreek (*Trigonella foenum-graecum* L.) is a legume crop that is used as a spice in cooking and in small quantities is categorized as “Generally Recognized as Safe” by the U.S. Food and Drug Administration [11]. Due to its amazing therapeutic and medical properties it is one of the oldest medicinal plants known and has long been recognized as a traditional medicine in different parts of the world and in Egypt as well [12, 13]. Recent reports showed that the seeds of the plant have been used as an old-style medicine for abundant circumstances, because it contains different active chemical constituents, including steroidal sapogenins [14], dietary fiber [13], galactomannans [15], antioxidants, and amino acids such as 4-hydroxyisoleucine, which possess antidiabetic [16], hypocholesterolemic, and hypoglycemic properties [17] that show a potential treatment as antileukemic, antipyretic [18], and antifertility activity [19], obesity, diabetes, and cancer treatment [20, 21].

Flavonoids have high pharmacological action and excessive anxiety in these constituents has been stimulated by the prospective health benefits arising from the antioxidant activity of these polyphenolic compounds [22]. The seed extract contains another bioactive compound, which is called diosgenin that induces apoptosis in HT-29 human colon cancer cells [23]; besides it plays a role in osteoclastogenesis, invasion, and proliferation inhibition through the downregulation of Akt, I kappa B kinase activation, and NF kappa B-regulated gene expression in tumor cells [24]. Furthermore, some constituent of alkaloids, “trigonelline,” has a potential role in cancer treatment [25]. Another active agent identified in Fenugreek is Protodioscin, which induces apoptosis in the leukemic cell line HL-60 [26].

The prototypic molecular change associated with cancer is mutation of tumor suppressor* p53*, which is inactivated in 50% of human cancers [27]. The p53 protein is a key regulator of cell cycle arrest and apoptosis and is controlled by a complex network of posttranslational modifications that regulate its activity, stability, and molecular interactions [28]. In HCC,* p53* mutation occurs late in hepatocarcinogenesis [29]. However, the relationship between* p53*WT expression and differentiation grade has not been well described. Intervention to restore wild type* p53* activity is an alternative approach for HCC treatment. Recently, many studies from our laboratory and others focused on p53 role in the pathogenesis and development, diagnosis and treatment, and therapeutic effects and prognosis of HCC [30–32].

Proliferating cell nuclear antigen (PCNA), an essential regulator of the cell cycle, is a 36 kDa molecule, which is highly conserved among species. PCNA is an evolutionally well-conserved protein found in all eukaryotic species from yeast to humans, as well as in archaea. Its functions are related to vital cellular processes such as DNA replication, chromatin remodeling, DNA repair, sister chromatid cohesion, and cell cycle control [33]. PCNA role and interactions are modulated by posttranslational regulation, whose exact mechanisms are controversial and not completely understood. Many reports showed posttranslational regulation of PCNA modifications, including [34] phosphorylation, acetylation [35, 36], and methyl esterification [37]. In addition, PCNA is important to determine its role in proliferative activity in different tumors including HCC [38].

The present study was aimed at evaluating the therapeutic effect of Fenugreek crude extract (FCE) against immortalized HCC cell line, HepG2. In addition, we focused on the role of FCE on p53, Bax, and PCNA protein expression levels as one of the key proteins involved in apoptosis induction in HepG2 cell line.

## 2. Materials and Methods 

### 2.1. Plant Material and Extraction Procedure

*Trigonella foenum*-*graecum* seeds were bought fresh from local market, Alexandria, Egypt, and the purity and quality of the seeds were investigated by Drs. M. M. Ibrahim and G. A. El-Gaaly who are experts in this field. The air-dried seeds were ground multiple times with an electric grinder. 15 gm of the power-driven seeds was weighted, transferred to flask, treated with the methanol until the powder was fully immersed, and refluxed for 24 h at 50°C. The resulting supernatant was filtered and evaporated. The seed residue was soaked and refluxed with 1 liter each of hexane, petroleum ether, ethyl acetate, and chloroform, respectively. The resulting filtered supernatants were combined and evaporated.

### 2.2. GC-MS Analysis

The GC-MS analysis was carried out using a Clarus 500 PerkinElmer (Auto system XL) Gas Chromatograph equipped and coupled to a mass detector Turbo Mass Gold, PerkinElmer Turbo Mass 5.1 spectrometer with an Elite-1 (100% dimethylpolysiloxane), 30 m × 0.25 mm ID × 1 *μ*m of capillary column. The instrument was set to an initial temperature of 110°C and maintained at this temperature for 2 min. At the end of this period, the oven temperature was rose up to 280°C, at the rate of an increase of 5°C/min, and maintained for 9 min. Injection port temperature was ensured as 250°C and helium flow rate as one mL/min. The ionization voltage was 70 eV. The samples were injected in split mode as 10 : 1. Mass spectral scan range was set at 45–450 (*m*/z).

### 2.3. Identification of Phytocompounds of FCE

Using the database of Wiley 275 L. library and comparing the spectrum obtained through GC-MS compounds present in the plants sample were identified. Interpretation on mass-spectrum GC-MS was conducted using the database of Wiley 275 L. The spectrum of the unknown components was compared with the spectrum of known components stored in the Wiley 25 L. library. The name, molecular weight, and structure of the components of the test materials were ascertained.

### 2.4. Cell Culture

HepG2 cells were obtained from American Type Culture Collection (Rockville, MD, USA) and maintained in Dulbecco's modified essential media (DMEM, Gibco) supplemented with 10% Fetal Bovine Serum (FBS), 100 Units/mL penicillin, and 100 *μ*g/mL streptomycin at 37°C in a 5% CO_2_ atmosphere.

### 2.5. Cell Cytotoxicity (MTT Assay) of HepG2 Cell Line

HepG2 cells were cultured as described above till mid-log phase. Cells were harvested and resuspended in growth media to make a stock cell suspension containing 20,000 cells/mL. 100 *μ*L of this stock cell suspension was added to the wells of a 96-well plate. The cells were allowed to attach and grow for 24 h. The crude extract was weighed and diluted with DMSO to make a 100 mg/mL stock solution. This stock solution was further diluted with culture media to make a secondary working solution. The working solution was added to the wells such that final concentrations of range 0~2000 *μ*g/mL of FCE were obtained. Each experiment was performed in triplicate in parallel for each concentration. Controls were performed in which only culture media and DMSO were added. The cells were then incubated at 37°C in a 5% CO_2_, 95% air atmosphere. After 72 h of incubation, the culture medium was removed and the cells were washed twice with phosphate buffered saline (PBS). Then 20 *μ*L of 5 mg/mL MTT [3-(4,5-dimethylthiazol-2-yl)-2,5-diphenyltetrazolium bromide] was added to each well. The cells were further incubated at 37°C for 4 h. The supernatant was discarded and 100 *μ*L of DMSO was added to each well. The mixture was shaken on a microvibrator for 5 min and the absorbance was measured at 570 nm (*A*) that served as a measure of cell viability. Inhibition ratio (*I*%) was calculated using the following equation: Formula *I*% = (*A*_control_ − *A*_treated_)/*A*_control_ × 100.

### 2.6. Morphological Analysis

Morphological observation of HepG2 cells treated with FCE was done to determine the changes induced by treatment. All the cells were exposed to increasing concentrations (100~500 *μ*g/mL range) of FCE for 48 h and cell images were taken using an inverted phase contrast microscope at 200x magnification.

### 2.7. Apoptosis Enzyme-Linked Immunosorbent Assay

Cells were seeded at a density of 2 × 10^4^/well in a 96-well plate and incubated for 24 h. Media were changed to media containing the tested extract (100~500 *μ*g/mL range) dose. Cells were then incubated for extra 24 h. An ELISA assay was performed, using Cell Death Detection ELISAPLUS kit (cat. number 11774425001) (Roche-Applied Science, Indianapolis, USA) that measures histone release from fragmented DNA in apoptotic cells. The assay is based on the quantitative “sandwich enzyme immunoassay” principle using mouse monoclonal antibodies directed against DNA and histones. This allows the specific determination of mono- and oligonucleosomes in the cytoplasmic fraction of cell lysates. Cells were lysed with 200 *μ*L lysis buffer for 30 min at room temperature. The lysate was subjected to centrifugation for 10 min and 200 *μ*L of supernatant was collected, of which 20 *μ*L was incubated with anti-histone biotin and anti-DNA peroxidase at room temperature for 2 h. After washing with incubation buffer three times, 100 *μ*L of substrate solution (2,2′-azino-di(3-ethylbenzthiazoline-sulphuric acid)) was added to each well and incubated for 15–20 min at room temperature. The absorbance was measured using an ELISA reader (Jenway Spectrophotometer, UK). Each assay was done in triplicate and standard deviation was determined.

### 2.8. Flow Cytometric Analysis

Cells were seeded at a density of 3–5 × 10^5^/10-cm^2^ plate and incubated for 24 before treatment. Media were changed to media containing 100~500 *μ*g/mL range of FCE for 48 h. Cells were harvested by trypsinization, washed with PBS, and fixed with ice-cold 70% ethanol while vortexing. Finally, the cells were washed and resuspended in PBS containing 5 *μ*g/mL RNase-A (Sigma, St. Louis, MO, USA) and 50 *μ*g/mL propidium iodide (Sigma, St. Louis, MO, USA) for cell cycle analysis. Cell cycle analysis was performed, using FACScan Flow Cytometer (Becton Dickson) according to the manufacturer's protocol.

### 2.9. Caspase-3 Activity

Caspase-3 activity was assayed according to manufacturer's protocol of colorimetric assay kit (BioVision, Inc., CA, USA) that provides a quick and efficient detection of caspase-3 activity in cell lysates and in purified preparations of caspase-3. 5 × 10^6^ cells were treated with or without 100~500 *μ*g/mL FCE and lysed in 100 *μ*L lysis buffer containing 10 mM HEPES (4-(2-hydroxyethyl)-1-piperazineethanesulfonic acid), pH 7.4, 2 mM EDTA, 0.1% CHAPS (3-[(3-cholamidopropyl)dimethylammonio]-1-propanesulfonic acid, 5 mM, 350 *μ*g/mL PMSF (phenylmethanesulfonylfluoride or phenylmethylsulfonyl fluoride), and 5 mM DTT (Dithiothreitol). Cells were homogenized by three cycles of freezing and thawing and then centrifuged to remove the cellular debris. Each sample was then incubated in buffer containing 10 mM HEPES, pH 7.4, 2 mM EDTA, 0.1% CHAPS, and 5 mM EDTA supplemented with its substrate (Ac-Asp-Glu-Val-Asp-AFC) Ac-DEVD-AFC for 1 h at room temperature and then reaction was stopped with 1 N HCl. OD405 was measured using a spectrophotometer (Jenway Spectrophotometer, UK). Each assay was done in triplicate and standard deviation was determined.

### 2.10. Western Blotting Analysis

Cells were plated in 6-well dishes for western blotting analysis. Cells were lysed with a 1% NP-40 containing buffer supplemented with a 1x cocktail of protease inhibitors (Complete Mini, Roche, Mannheim, Germany) and phosphatase inhibitors (phosphatase inhibitor cocktail I and II, Sigma) at 4°C for 30 min. Lysates were centrifuged at 10,000 g at 4°C for 15 min and supernatants collected. The protein concentration of the supernatant was determined using the BCA assay (Pierce, Rockford, IL). Samples were mixed in a ratio of 1 : 2 in Laemmli buffer and denatured by heating at 98°C for 5 min. Forty *μ*g of protein was separated on 10% Tris-SDS-PAGE gels (Bio-Rad Laboratories, Hercules, CA, USA) at 100 V for 1 h. For western blotting, the separated proteins were electrophoretically transferred onto polyvinylidene difluoride membranes (Bio-Rad Laboratories, Hercules, CA, USA) at 380 mA for 1 h. Western blot analysis was carried out using specific primary antibodies for p53 and Bax (Santa Cruz Biotechnology, CA, USA) and PCNA and anti-poly(ADP-ribose) polymerase (Cell Signaling Technology, Beverly, MA, USA) antibodies. The expression of *β*-actin (Sigma-Aldrich, St. Louis, MO, USA) was used as a normalization control for protein loading. The membranes were blocked with TBS plus 5% nonfat milk (20 mM Tris-HCl, pH 7.6, 137 mM NaCl) followed by incubation overnight with primary antibodies diluted in blocking solution for antibodies (1–1000). This was followed by incubation again for 1 h in the appropriate horseradish peroxidase-conjugated secondary antibodies (Santa Cruz Biotechnology, CA, USA). For detection, an ECL kit was used according to the manufacturer's instructions (Amersham, Buckinghamshire, UK). The corresponding relative density of p53 and PCNA bands was calculated by Quantity One software.

## 3. Results

### 3.1. Effects of FCE on the Inhibition of HepG2 Cell Proliferation

**Figure 1:**
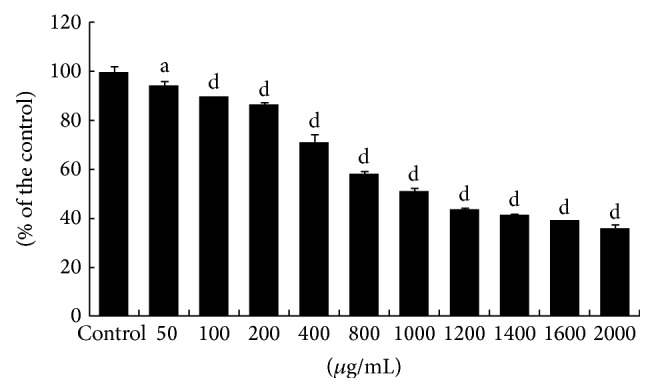
Effect of Fenugreek* seed crude extract (FCE)* on cell viability of HepG2 cells. MTT assay was performed to detect the living cells as described under Section 2. Each data point is an average of results from three independent experiments performed in triplicate and presented as M ± SD.

Exponentially growing HepG2 cell line was cultured continuously in the absence or presence of different concentrations of FCE. The effects of tested FCE on cell growth were assessed by the MTT assay after 48 h of incubation with FCE as described under Section 2. The concentration of 50% inhibition of HepG2 cell viability was calculated as IC_50_, using a Semilogarithmic plotting of the % of cell viability versus concentration of the tested extract. The (IC_50_ = 1000 *μ*g/mL) dose of FCE showed a significant decrease in cell survival of HepG2 cells compared to control group at 48 h (Figure 1). As shown in Figure 1, treatment by concentrations range of 0~2000 *μ*g/mL for 48 h of FCE showed a significant dose-dependent cytotoxic effects on HepG2 cell line with complete elimination of all cells at a dose over 1000 *μ*g/mL (Figure 1). Our data showed that FCE significantly decreases HepG2 cell viability in dose-dependent manner. Significant decrease of cell viability was observed at 100 *μ*g/mL and above concentrations of FCE compared to control untreated group (*P* < 0.01, unpaired *t*-test).

### 3.2. Morphological Changes

**Figure 2:**
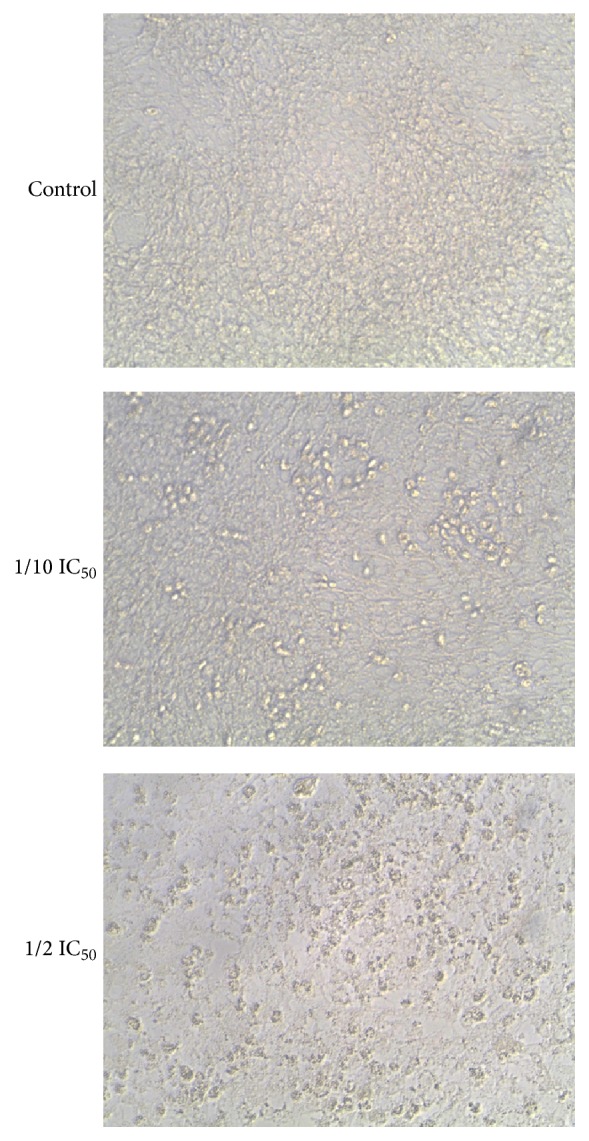
Fenugreek* seed crude extract (FCE) induced changes in HepG-2 cell line morphology.* The results revealed that the control cells showed a typical polygonal and intact appearance, whereas the 100~500 *μ*g/mL range of FCE-treated cells displayed morphological changes with preapoptotic characteristics. Representative data from three independent experiments are shown, using inverted phase contrast microscope at 200x magnification.

The morphological changes observed in HepG2 treated with or without FCE treatment for 48 h are shown in Figure 2. Morphological alterations of HepG2 cell line after (100~500 *μ*g/mL) FCE treatment for 48 h were observed under phase contrast inverted microscope. The cells indicated the most prominent effects after FCE treatment starting at 24 h~48 h. Changes in morphology were found in concentration-dependent manner. Cells exposed to concentration range of 100~500 *μ*g/mL of FCE for 48 h altered the normal morphology of the cells and cell adhesion capacity in comparison to control (Figure 2). The effect of FCE on treated cells started at 100 *μ*g/mL in which the cells lost their typical morphology and appeared smaller in size, shrunken, and rounded. Furthermore, treatment of the cells with the same concentrations for 72 h killed most of the cells (data not shown).

### 3.3. Apoptosis Enzyme-Linked Immunosorbent Assay, ELISA

**Figure 3:**
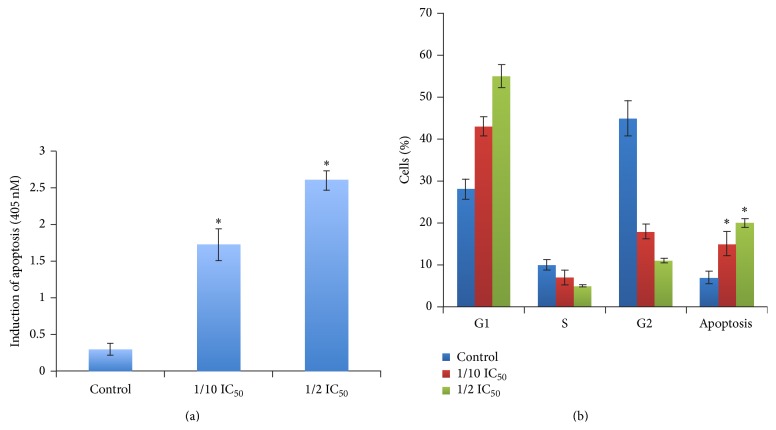
(a)* Apoptosis enzyme*-*linked immunosorbent assay, ELISA.* Apoptosis detection assay was applied to detect apoptosis induction in HepG2 cells after treatment with FCE. Cells were treated with (100~500 *μ*g/mL range) FCE for 48 h. Cells without drug treatment were used as controls. ^∗^ *P* < 0.05 as compared to the untreated controls, using the unpaired Student *t*-test. Each data point is an average of three independent experiments and is expressed as M ± SD. (b)* Flow cytometric analysis of HepG2 cells after treatment with or without* Fenugreek* seed crude extract (FCE).* Cell cycle analysis shows % of cell cycle phases of HepG2 cell line treated with or without 100~500 *μ*g/mL range of FCE for 48 h. ^∗^ *P* < 0.05 as compared to the untreated controls, using the unpaired Student *t*-test. Each data point was an average of results from three independent experiments performed in triplicate and presented as M ± SD.

Our data indicated that FCE significantly decreased cell viability compared to control cells as shown in Figure 1. It is possible that the decrease in cell viability by FCE treatment as determined by MTT assay could be due to either cell growth arrest or cell death. Understanding the molecular mechanism associated with hepatocellular carcinoma improves screening and treatment of the disease [39]. To investigate if the decrease in cells viability could be due to apoptosis induction, apoptotic assay, using ELISA technique, was performed, which detected histone release from apoptotic cells (Figure 3(a)). HepG2 cells were treated with (100~500 *μ*g/mL) FCE and induced apoptosis was determined as indicated by histone release according to manufacturer's protocol compared to untreated, control cells; more details are under Section 2. In addition, cell death was assayed by Trypan blue staining, which determines membrane integrity (data not shown). FCE-induced cell death was a relatively late event, starting after 48 h of treatment with treatment by (100~500 *μ*g/mL) FCE. The number of both the Trypan blue-dead cells and the condensed and fragmented nuclei increased with increasing FCE concentration, confirming that FCE-induced cell death was due to apoptosis induction in HepG2 cells. Furthermore, we performed cell cycle analysis to examine and confirm the effect of FCE treatment on apoptosis induction in HepG2 cells for 48 h (Figure 3(b)). Consistent with the results of ELISA, cells were treated with or without (100~500 *μ*g/mL) FCE for 48 h. Our data revealed that a significant increase in apoptotic HepG2 cells at sub-G1 was detected by flow cytometry analysis and the ratio was ~34.9 and 49.1%, respectively, compared to the untreated control cells. Flow cytometry analysis also showed a significant increase in G1 phase through arresting cells by ratio of ~53.3 and 65%, respectively, compared to untreated cells as shown in Figure 3(b), indicating that FCE significantly induced apoptosis in HepG2 cells. Induced apoptosis by 500 *μ*g/mL FCE treatment showed more significant effects compared to 100 *μ*g/mL FCE treatment.

### 3.4. Caspase-3 Activity

**Figure 4:**
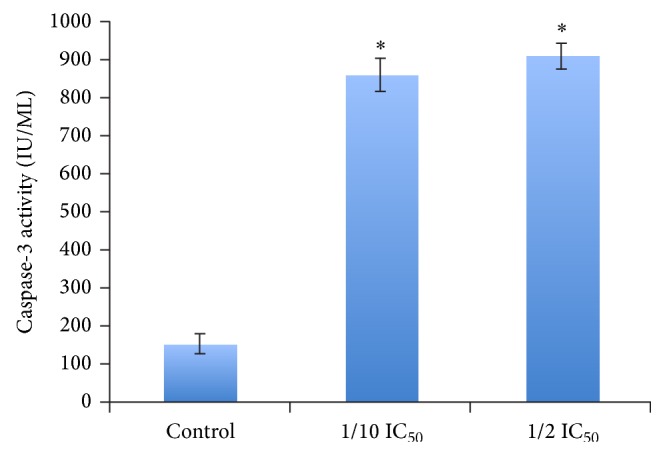
*Effect of* Fenugreek* seed crude extract* (*FCE) on caspase-3 activity (as indicated by (DEVD-pNA) cleavage) of HepG2 cells.* Cells were treated with or without 100~500 *μ*g/mL range of FCE for 48 h. Cells without drug treatment were used as controls. ^∗^ *P* < 0.05 as compared to the untreated controls, using the unpaired Student *t*-test. Each data point is an average of three independent experiments and is expressed as M ± SD. More details are under Section 2.

One of the key events in apoptosis is the activation of a cascade of intracellular cysteine proteases known as caspases [40]. On proteolytic activation by upstream caspases, caspase-3 is able to cleave a variety of substrates, including poly(ADP-ribose) polymerase (PARP). The cleavage of various substrates contributes to the typical morphological and biochemical features observed in apoptosis. Because of the diversity of its substrates, caspase-3 is thought to be a general mediator of physiological and stress-induced apoptosis [41, 42]. To investigate if the FCE could be able to activate caspase-3 in treated HepG2 cells, caspase-3 activity was assayed according to manufacturer's protocol (more details are under Section 2) (Figure 4). Our data indicated that the FCE showed a significant increase in caspase-3 activity in treated HepG2 cells if compared with untreated cells after 48 h. As shown in Figure 4, the FCE at the apoptosis-inducing concentration (100~500 *μ*g/mL) dose strongly stimulated caspase-3-like activity in HepG2 cells. These results confirmed that FCE-induced apoptosis of HepG2 cells was in a caspase-dependent manner.

### 3.5. Western Blot Analysis

**Figure 5:**
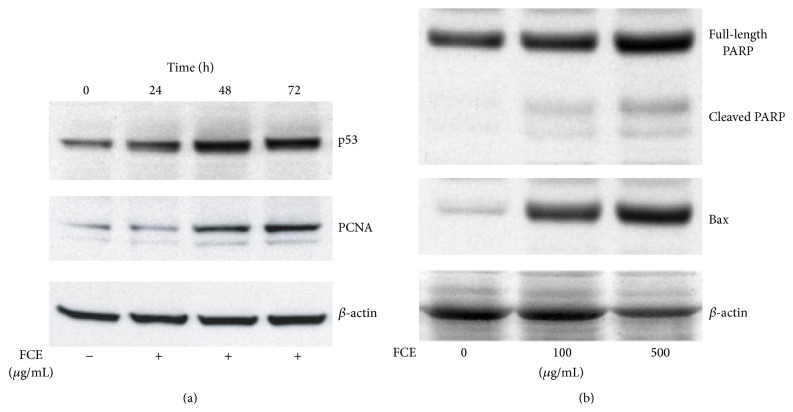
(a) Fenugreek* seed crude extract (FCE)* upregulated p53 and PCNA expression levels in HepG-2 cells line, using western blot analysis. Cells were treated with or without (500 *μ*g/mL) FCE for different time intervals. Our data showed a significant increase in p53 and PCNA protein expression, respectively. Blots were normalized for total protein loading. Representative data from three independent experiments are shown. (b) Apoptosis indicated by poly(ADP-ribose) polymerase (PARP) cleavage. Western blot analysis for poly(ADP-ribose) polymerase (upper panel) and Bax protein expression (lower panel). Cells were treated with or without 100–500 *μ*g/mL range of Fenugreek seed crude extract, FCE, for 48 h. Blots were normalized for total protein loading.

To investigate the intracellular mechanism for the observed increase in apoptosis in FCE-treated HepG2 cells, the protein expression levels of the p53, Bax as a well-known tumor suppressor protein and proliferating cell nuclear antigen (PCNA), an essential regulator of the cell cycle, were examined. The p53 tumor suppressor protein is a key regulator of cell cycle arrest and of apoptosis. During DNA damage, the p53 is normally activated and accumulated to exert its DNA-binding activity for the regulation of different genes that are involved in cell cycle regulation and apoptosis induction. To understand the mechanism behind the decreased cell viability after FCE treatment, we examined the p53 protein expression for different time intervals. Western blot data showed that p53 protein expression was upregulated after (500 *μ*g/mL) FCE treatment for 48 h compared to untreated control cells (Figure 5(a)) and this expression was sustained till 72 h of treatment, suggesting that FCE treatment triggered p53 protein expression and induced apoptosis in HepG2 cells.

As PCNA is known to be a key regulator of cell cycle progression, we investigated the protein expression levels of PCNA with or without (500 *μ*g/mL) FCE treatment for different time intervals. The PCNA serves as a cofactor for DNA polymerase delta in S-phase and is involved in DNA repair during DNA synthesis [14, 43]. The temporal pattern of PCNA protein expression was upregulated and that is consistent with p53 expression pattern after (500 *μ*g/mL) FCE treatment for 48 and 72 h compared to untreated control cells (Figure 5(a)). PCNA was found valuable in studying the proliferative activity in different tumors including HCC [17, 34]. Our data give clear evidence that the proliferation activity and apoptosis induction are altered by FCE treatment in HepG2 cell line. Furthermore, western blot analysis showed that the occurrence of apoptosis was confirmed by cleavage of poly(ADP-ribose) polymerase most notably at 48 h after (100, 500 *μ*g/mL) FCE treatment, which is consistent with induced significant growth inhibition (Figure 5(b); upper panel).

One of proapoptotic genes is the Bax gene that has been found to be a transcriptional target of p53, which was also determined by western blot analysis. Our data indicated that (100, 500 *μ*g/mL) FCE treatment for 48 h upregulated Bax expression by 3.1- and 3.3-fold, respectively, compared to control cells (Figure 5(b), lower panel). Taken together, our data indicated that FCE treatment induced apoptosis in HepG2 cells via arresting cells in G1 phase of cell cycle, upregulating p53 and proapoptotic gene, Bax, and expression levels, and increasing caspase-3 activity.

### 3.6. GC-MS Analytical Data

**Figure 6:**
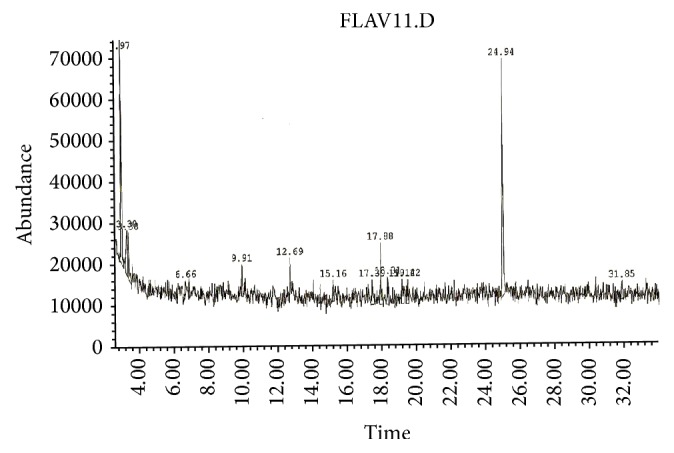
GC-MS chromatogram of methanol extract of* Trigonella foenum*-*graecum,* Fenugreek, FCE.

GC-MS analysis of the bioactive constituents of the methanol extracted* Trigonella foenum-graecum* seeds (FCE) clearly showed the presence of fourteen compounds and their chromatogram is shown in Figure 6. Major peaks were determined in GC-MS chromatogram of FCE and their corresponding components. The active principles with their retention time (RT), molecular formula, molecular weight (MW), and concentration (peak area %) are presented in Table 1. From the above study it may be concluded that* Trigonella foenum*-*graecum* extract contains many important phytochemicals, such as Flavonoids and Terpenoids, which play an effective role as pharmaceutical anticancer agents.

## 4. Discussion

Despite the combined efforts of governments and scientists worldwide, there is constant increase in the incidence of hepatocellular carcinoma during the last two decades [44]. Successful HCC treatment requires an adequate therapeutic index reflecting the treatment's specific effects on target cells and its lack of clinically significant effects on the host. Fenugreek is commonly used as a spice in food preparations due to the strong flavor and aroma and is used in traditional medicines as leads for therapeutic drug development in modern medicine. Pharmacological features of Fenugreek seed are known such as hypoglycaemic, hypercholesterolaemic, gastroprotective, and hepatoprotective activity [45, 46] and antioxidant properties against experimental cataract [47].

In the present study, HepG2 cell line was selected to investigate the cytotoxicity effect of FCE on HCC. Herein, we demonstrated the cytotoxicity effect of FCE by measuring the percentage of cell mortality that was calculated by MTT assay and apoptosis induction in the HepG2 cells at different time intervals of treatment. Our data showed that FCE significantly reduced the viability of HepG2 cells in a concentration-dependent manner. The highest decrease in the cell viability of HepG2 cells was in treated cells by the 1/2 IC_50_ for 48 h. Our data agreed with the previous findings where they found difference in the sensitivity of the cell lines [48]. The reduction in percentage of cell viability after 48 h of treatment showed potent cytotoxic effects on HepG2 cells with FCE treatment and this effect sustained for 72 h. Our results are also in well correlation with the previous findings in which the extract was found more cytotoxic to cancerous cells than normal cells [49] due to the sensitivity of cancerous cells towards the death flavanoids [50]. The morphological changes in the HepG2 cells were observed more prominent in treated cells showing extensive blebbing and vacuolation, suggesting autophagic mechanism of cell death [51]. At 48 h of FCE treatment, HepG2 cells began to display apoptotic morphology, including shrinkage, DNA condensations, nuclear and plasma membrane convulsion, enucleation and apoptotic bodies formation, which can be seen as small masses, and nuclear fragmentation.

Modulation of the p53 gene is frequently a genetic change in different human cancers. Over 50% of all human malignancies contain mutations of p53 gene [27]. p53 has been reported to play a role in transcriptional activator to induce the transcription of many genes, including apoptosis-related genes. One of proapoptotic genes is Bax gene that has been found to be a transcriptional target of p53 and could be upregulated in response to a variety of p53-dependent apoptosis triggers [52]. Our data demonstrates that HepG2 cells treated by FCE showed a significant increase in the protein expression levels of p53 and Bax after 48 h, suggesting that Bax could be a transcriptional target of upregulated p53 which in turn could be responsible for inhibition of cell viability, apoptosis induction, and regulation of cell cycle related p53 genes. Previous studies also showed that diosgenin, one of the FCE active ingredients, induced apoptosis in HT-29 human colon cancer cells [23]. Furthermore, it was reported that the antiproliferative effect of diosgenin through activation of p53 could be by releasing of apoptosis-inducing factor (AIF) and modulation of caspase-3 activity in different human cancer cells [52].

In prostate cancer cells, PC-3, a cell line expressing wild type p53, FCE induced p21 expression that correlated with a cytotoxic response, suggesting a proapoptotic role for induced p21 [53]. In contrast, FCE effects are varied in different cell lines. For instance, in the mutant p53 expressing cell lines, HepG3, FCE downregulated p53 expression [54]. Furthermore, consistent with GC-MS analysis of the bioactive constituents of the methanolic extract of FCE, it was reported that FCE-induced apoptosis was mediated by the death receptor pathway, suggesting presence of some phytocompounds in FCE extract [55]. HepG2 cells express a wild type and an inducible p53 activity, as well documented previously [56], which in turn could explain the involvement of wild type upregulation in the FCE-induced apoptosis in HepG2 cell line.

Proliferating cell nuclear antigen (PCNA) is a 36 kDa protein involved in several cellular mechanisms, including DNA synthesis and repair, cell cycle regulation, and apoptosis. An alteration in PCNA structure might contribute to DNA-damage accumulation in cancer cells. The present study was aimed at evaluating the PCNA expression pattern in HCC cell line, HepG2, after FCE treatment for different time intervals. Our data showed that PCNA protein expression was significantly increased with FCE treatment for 48 and 72 h, respectively. The PCNA upregulation data was consistent with p53 upregulation pattern for the same period of treatment. Both by p53-dependent and -independent regulations, PCNA interacts with multiple proteins that play a key role in DNA synthesis and repair, cell cycle regulation, chromatin remodeling, and apoptosis [57].

Alterations of p53 protein have been observed during hepatocarcinogenesis [58], and the overexpression of p53 correlates with a high level of proliferation of cell nuclear antigen (PCNA), HCC dedifferentiation, and advanced HCC stages [59]. Our data showed that the treatment of HepG2 with 1/2 IC_50_ of FCE (500 g/mL) for 24 and 72 h exhibited an upregulation in the expression of both p53 and PCNA compared to the untreated control. It was expected that PCNA expression would rather be downregulated with the upregulation of p53. However, very recently, in agreement with our data, the naturally occurring selenomethionine (SeMet) upregulated the expression of p53 and PCNA and induced base excision repair (BER) activity. The distinct chemopreventive mechanism is p53-dependent and involves the binding of Gadd45a and two BER-mediated repair proteins, PCNA and apurinic/apyrimidinic endonuclease (APE1/Ref-1) [60]. Furthermore, it was reported that PCNA gene is induced by p53, while PCNA protein interacts with p53-controlled proteins Gadd45, MyD118, CR6, and p21, in the process of deciding cell fate [61]. The binding partner of Gadd45a can decide the activity that mediates, in other words, the binding of PCNA and p21^WAF1/CIP1^ or Cdc2 to Gadd45a can mediate DNA repair or cell cycle arrest, respectively. In addition, the possibility that p53 may be involved in the induction of human PCNA was reported earlier; the human PCNA promoter has a p53 binding site and is transactivated by wild type p53 which in turn leads to DNA repair [62]. In contrast, if PCNA is slightly upregulated in cell, apoptosis also occurs [63]. The dual role of PCNA in HepG2 cells could be explained by the presence of acidic and basic PCNA isoforms, according to previous reports [64, 65]. The isoforms having a basic pI were exclusively found in malignant cells. The PCNA basic isoforms, exclusively detected in hepatic malignant samples, may represent a new signature for neoplastic liver cells compared to cirrhotic tissues. This finding revealed that PCNA is differently expressed in HCC, in terms of structure, isoforms, and posttranslational modifications, implicating a role for PCNA functional alterations in hepatocarcinogenesis process.

### 4.1. GC-MS Analytical Data

GC-MS analysis of the bioactive constituents of the methanolic extract of FCE clearly showed the presence of fourteen bioactive compounds. Our results revealed that Squalene and Naringenin were detected as the two main components in the methanol extract of 27.71% and 24.05%, respectively. In addition, the rest of components were ranged among 1.87% for butane-2,3-diol, 6.98% for 2-propen-1-amine, N-ethyl- and aziridine, and 1,2,3-trimethyl-trans (6.62%). Our results also reveal the presence of several fatty acids; in addition to many Flavonoids compounds were identified in GC-MS analysis of the* Trigonella foenum* seeds, including major components such as Tricin and its derivatives. Many compounds from our GC-MS analysis have anticancer activity such as Naringenin and Squalene.

Naringenin, a flavonoid that is present in high concentrations in grapefruits and citrus fruits, has a wide spectrum of pharmacological activities, including anticancer activity in primary tumor prevention [66, 67]. Furthermore, Squalene and Quercetin appear to influence several biochemical and physiological activities, which are intriguing for the treatment of cancer. Quercetin modulates the pathway toward suppression of lymphoma by downregulating PI3K-AKT1 and upregulating p53 pathways as well as by glycolytic metabolism [68].

## 5. Conclusion 

GC-MS analysis of methanol extract of* Trigonella foenum*-*graecum*, Fenugreek* seed crude extract (FCE),* contains many important phytochemical, such as several fatty acids, in addition to many Flavonoids compounds, Tricin and its derivatives. Many other major compounds from our GC-MS analysis have anticancer activity such as Naringenin, Quercetin, and Squalene.

The mechanism of FCE-induced cell death was via inhibiting HepG2 cell viability and inducing apoptosis. FCE enhances apoptosis induction in HepG2 cell in p53, Bax and PCNA-dependent pathway, and G1 phase arrest that was confirmed by cell cycle analysis. Despite the finding of p53 involvement in FCE cell viability inhibition and apoptosis induction, the exact downstream target of p53 is unclear, which requires further investigation. However, we suggest that induction of p53, Bax, and PCNA-dependent pathway which mediated apoptosis with caspase-3 activation is, at least in part, a possible explanation for the anti-liver-carcinogenic effect of FCE which in turn might be a potential strategy for HCC treatment.

## Figures and Tables

**Table 1:** Total ionic chromatogram (GC-MS) of methanol extract of *Trigonella foenum-graecum, *Fenugreek, obtained with 70 eV, using Elite-1 fused silica capillary column with helium gas as the carrier.

PK	RT	Peak area	Name of the compound	Molecular formula	Molecular weight	Category of the compound
1	2.98	24.05	Naringenin	C_15_H_12_O_5_	272	Flavonoids
2	3.29	6.62	Obtusifolin (anthraquinone)	C_5_N_11_N	85	Glycosides
3	3.38	6.98	Tricin	C_17_N_14_O_7_	330	Flavonoids
4	6.66	1.87	Vitexin	C_21_H_20_O_10_	432	Flavonoids
5	9.91	3.06	Quercetin	C_15_H_10_O_7_	330	Flavonoids
6	12.69	3.04	Limonene	C_10_H_16_	136	Terpenoids
7	15.16	2.33	Heptanoic acid	C_9_H_18_O_2_	158	Bisphenol
8	17.40	3.51	Tricine	C_6_H_13_NO_5_	179	Flavonoids
9	17.87	6.77	Kaempferol	C_10_H_16_O	152	Flavonoids
10	18.32	4.04	*γ*-Terpinene	C_12_H_16_	132	Terpenoids
11	19.13	3.51	Carvone	C_10_H_14_O	152	Terpenoids
12	19.42	4.09	1-Tridecyne	C_13_H_24_	180	Flavonoids
13	24.94	27.71	Squalene	C_30_H_50_	410	Terpenoids
14	31.84	2.42	*α*-Linolenic acid	C_19_H_32_O_2_	292	Fatty acids
